# CRABP1 is associated with a poor prognosis in breast cancer: adding to the complexity of breast cancer cell response to retinoic acid

**DOI:** 10.1186/s12943-015-0380-7

**Published:** 2015-07-05

**Authors:** Rong-Zong Liu, Elizabeth Garcia, Darryl D. Glubrecht, Ho Yin Poon, John R. Mackey, Roseline Godbout

**Affiliations:** Department of Oncology, University of Alberta, Cross Cancer Institute, 11560 University Avenue, Edmonton, T6G 1Z2 AB Canada

**Keywords:** Cellular retinoic acid binding protein 1, Retinoic acid resistance, Retinoic acid receptor, Triple-negative breast cancer, Prognosis

## Abstract

**Background:**

Clinical trials designed to test the efficacy of retinoic acid (RA) as an adjuvant for the treatment of solid cancers have been disappointing, primarily due to RA resistance. Estrogen receptor (ER)-negative breast cancer cells are more resistant to RA than ER-positive cells. The expression and subcellular distribution of two RA-binding proteins, FABP5 and CRABP2, has already been shown to play critical roles in breast cancer cell response to RA. CRABP1, a third member of the RA-binding protein family, has not previously been investigated as a possible mediator of RA action in breast cancer.

**Methods:**

CRABP1 and CRABP2 expression in primary breast tumor tissues was analyzed using gene expression and tissue microarrays. CRABP1 levels were manipulated using siRNAs and by transient overexpression. RA-induced subcellular translocation of CRABPs was examined by immunofluorescence microscopy and immunoblotting. RA-induced transactivation of RAR was analyzed using a RA response element (RARE)-driven luciferase reporter system. Effects of CRABP1 expression and RA treatment on downstream gene expression were investigated by semi-quantitative RT-PCR analysis.

**Results:**

Compared to normal mammary tissues, CRABP1 expression is significantly down-regulated in ER+ breast tumors, but maintained in triple-negative breast cancers. Elevated CRABP1 levels are associated with poor patient prognosis, high Ki67 immunoreactivity and high tumor grade in breast cancer. The prognostic significance of CRABP1 is attributed to its cytoplasmic localization. We demonstrate that CRABP1 expression attenuates RA-induced cell growth arrest and inhibits RA signalling in breast cancer cells by sequestering RA in the cytoplasm. We also show that CRABP1 affects the expression of genes involved in RA biosynthesis, trafficking and metabolism.

**Conclusions:**

CRABP1 is an adverse factor for clinical outcome in triple-negative breast cancer and a potent inhibitor of RA signalling in breast cancer cells. Our data indicate that CRABP1, in conjunction with previously identified CRABP2 and FABP5, plays a key role in breast cancer cell response to RA. We propose that these three RA-binding proteins can serve as biomarkers for predicting triple-negative breast cancer response to RA, with elevated levels of either cytoplasmic CRABP1 or FABP5 associated with RA resistance, and elevated levels of nuclear CRABP2 associated with sensitivity to RA.

**Electronic supplementary material:**

The online version of this article (doi:10.1186/s12943-015-0380-7) contains supplementary material, which is available to authorized users.

## Introduction

Current clinical management of breast cancer relies on clinicopathological features as well as expression of biological markers such as estrogen receptor (ER), progesterone receptor (PR) and epidermal growth factor receptor 2 (HER2) [1, 2]. While tamoxifen has been shown to be highly effective for the treatment of ER/PR-positive breast cancers [3], there are no specific molecular targets for tumors that don’t express ER, PR or HER2. These triple-negative tumors, which constitute 15–20 % of breast cancers [4], are more aggressive and less responsive to standard treatment than the more common ER/PR-positive breast cancers, and have a poorer prognosis [5, 6].

RA and its derivatives, collectively called retinoids, inhibit growth and induce apoptosis in a variety of epithelial cancer cells and hold great promise as chemotherapeutic agents [7–9]. Retinoids inhibit mitogen signalling [10] and induce downstream signalling pathways implicated in growth arrest, apoptosis and differentiation of precancerous and cancer cells [11–15]. However, with the exception of acute promyelocytic leukemia (APL) [14, 16, 17], clinical trials designed to test the efficacy of RA and its derivatives in the treatment of cancer have produced disappointing results, primarily because of RA-induced side effects and development of RA resistance [18, 19].

RA exerts its physiological effects by binding and activating nuclear retinoic acid receptors RARα, β and γ. RAR dimerizes with retinoid-X-receptor (RXR) and binds to retinoic acid response elements (RARE) in the promoters of target genes, regulating their transcription and function [7–9]. As intracellular transporters, the cellular RA binding proteins determine RA subcellular distribution, fate and function [20–22]. Two RA binding proteins, cellular retinoic acid binding protein 2 (CRABP2) and fatty acid-binding protein 5 (FABP5), have been shown to play opposing roles in mediating the RA cellular response by targeting RA to distinct nuclear receptors; i.e., delivery of RA to RARs by CRABP2 leads to inhibition of cell proliferation, whereas delivery of RA to peroxisome proliferator activated receptor beta (PPARβ) by FABP5 increases cell proliferation and causes RA resistance [23, 24].

We previously reported that FABP5 is preferentially expressed in ER- and triple-negative breast tumors [25], subtypes that are prone to RA resistance [26–28]. Furthermore, breast cancer cells with an elevated FABP5/CRABP2 ratio show increased resistance to RA [23, 25]. However, the FABP5/CRABP2 ratio does not always predict breast cancer cell response to RA, and RA resistance in the squamous cell carcinoma cell line COLO 16 cannot be overcome by either restoration of CRABP2 expression or an increased CRABP2/FABP5 ratio [29], indicating that other factors are involved.

Studies designed to examine the importance of CRABP1 in the clinical outcomes of various cancers have produced conflicting results [30–33]. Prior to this study, the expression and prognostic significance of CRABP1 in breast cancer had not been investigated. In light of CRABP1’s proposed role in attenuating RA activity by enhancing RA metabolism, expression of CRABP1 in breast cancer could have important implications for RA response. Here, we report the expression, clinicopathological association and function of CRABP1 in breast cancer. Our data indicate that CRABP1 is an adverse prognostic factor and a potent inhibitor of RA action in breast cancer which functions by sequestering RA in the cytoplasm rather than by enhancing RA metabolism. We propose that CRABP1 may serve as a biomarker to predict RA response and a target to optimize the efficacy of RA in breast cancer treatment.

## Materials and methods

### Chemicals, reagents and DNA constructs

All-trans retinoic acid was purchased from Sigma-Aldrich (Oakville, ON, Canada) and dissolved in DMSO (Sigma-Aldrich) at a concentration of 50 mM. Scrambled stealth siRNAs and gene-specific siRNAs targeting different regions of *CRABP1* mRNA (nucleotides 381–405 and 484–508 of GenBank mRNA sequence NM_004378) and *CRABP2* mRNA (nucleotides 418–442 and 465–489 of GenBank mRNA sequence NM_001878) were purchased from Life Technologies (Burlington, ON, Canada). The Lipofectamine RNAiMAX reagent (Life Technologies) was used for siRNA transfections. The pGL3-RARE-luciferase plasmid DNA was purchased from Addgene (Cambridge, MA, USA) and the luciferase assay system from Promega (Madison, WI, USA). Polyethylenimine (PEI) (Polysciences, Warrington, PA, USA) was used for plasmid DNA transfections. For gain-of-function studies, the entire open reading frame of CRABP1 was PCR-amplified and cloned into pcDNA3 (Life Technologies).

### Cell culture and siRNA transfection

ZR-75-1, MDA-MB-468, MDA-MB-435, BT-20, T47D, BT-474, MDA-MB-231, BT-483, MCF-7, SK-Br-3, BT-549 and Hs578T breast cancer cells were cultured in Dulbecco’s modification of Eagle’s medium (DMEM) supplemented with 10 % fetal calf serum, penicillin (100 units/mL) and streptomycin (100 μg/mL). Cells were grown at 37 °C in a humidified incubator with 5 % CO_2_. To knockdown CRABP1 and CRABP2, MCF-7 cells were transfected with 10 nM siRNA. The medium was replaced with fresh medium 18 h after transfection and the cells were cultured for an additional 48 h. Two rounds of siRNA transfections were performed for each experiment. Hs578T, BT-549 and SK-Br-3 cells were transfected with 7 μg of empty (control) or pcDNA3 expression construct (CRABP1 or CRABP2) as previously described [34]. For cell proliferation assays, 10,000 siRNA-transfected cells were seeded in each well of 12-well plates and cultured overnight in DMEM containing 10 % FBS. The medium was then replaced with FBS-supplemented medium containing the indicated concentrations of RA (or DMSO as a vehicle control). Five days later, cells were counted using a Coulter Particle and Size Analyzer (Coulter Corporation, Mississauga, Canada).

### Immunofluorescence analysis

MCF-7 cells were cultured on coverslips for 24 h and treated with 0.5 μM RA (dissolved in DMSO) or vehicle (DMSO) in serum-free DMEM medium for 6 h. Cells were then fixed in 1 % paraformaldehyde in PBS for 10 min and permeabilized in 0.5 % Triton X-100 for 5 min. Cells were immunostained with anti-CRABP1 or anti-CRABP2 antibodies, followed by Alexa 594-conjugated donkey anti-mouse (for CRABP1) or Alexa 555-conjugated donkey anti-rabbit (for CRABP2) secondary antibodies (Life Technologies). Images were acquired using a Zeiss LSM510 confocal microscope (Oberkochen, Germany) with a 40 ×/1.3 oil immersion lens.

### Patient population

A total of 176 treatment-naïve primary breast cancer samples and 10 normal breast tissue samples from reduction mammoplasties were obtained from the Canadian Breast Cancer Foundation Tumor Bank and used for gene expression microarray analysis as previously described [35]. Patient material and clinical information was collected under Research Ethics Board Protocol ETH-02-86-17. Tumor tissues were frozen and histologically analysed as previously described [35].

Patients received standardized guideline-based chemo- and hormone therapies: i.e., patients with ER-positive tumors received hormone therapy, those with HER2-positive tumors received trastuzumab, high-risk node-negative disease was treated with anthracycline chemotherapy whereas anthracycline plus taxane chemotherapy was used for the treatment of node-positive disease. The 176 patients selected for this study consisted of 88 patients who experienced early relapse (<5 years after the initial treatment) and 88 patients who had not relapsed. ER, PR and HER2 status, stage and time of follow-up were balanced between the two groups. The median follow-up time for surviving patients was 4.5 years. The gene profiling data used in this publication have been deposited in NCBI [GEO Datasets: GSE22820].

### RNA preparation, gene expression microarrays and RT-PCR

Total RNA was isolated from frozen human breast tumor biopsies using the TRIzol reagent (Life Technologies) and further purified with Qiagen RNeasy columns (Qiagen, Mississauga, ON, Canada). The average percent of area with tumor cells was 72.6 % and the average percent of cells that were tumor cells was 93.4 %, for all tissues analysed. Microarray hybridization was carried out as previously described [35]. Reverse transcription-polymerase chain reaction (RT-PCR) conditions were as previously described [25, 34]. The number of cycles for each primer pair was optimized for quantitative amplification within the exponential PCR product growth phase. PCR primers are listed in Additional file 1: Table S1. PCR amplification of human β-actin mRNA served as positive control as previously described [25]. For real-time quantitative RT-PCR (qRT-PCR), we used the following TaqMan FAM-labeled Gene Expression Assay primers: human CRABP1 (Hs00171635_m1), human CRABP2 (Hs00275636_m1) and human GAPDH (Hs03929097). cDNA samples were analysed in triplicate, with each cDNA undergoing 40 cycles of amplification in 96-well reaction plates (10 μL volume) using TaqMan Gene Expression Master Mix (Applied Biosystems 7900HT Real-Time PCR System).

### Generation of tissue microarrays and immunohistochemical (IHC) staining

Tissue microarrays (TMA) (TMArrayer, Pathology Devices) were generated using all available formalin-fixed paraffin-embedded breast tumor tissues representing 120 patients out of the 176-patient cohort used for gene expression analysis. The TMA slides contained triplicate core tissue samples (0.6 mm in diameter) from each tumor. TMAs were immunostained with anti-CRABP1 monoclonal antibody (Sigma-Aldrich; 1:200 dilution) and anti-CRABP2 polyclonal antibody (Protein Tech Group; 1:200 dilution). The signal was detected using EnVision + anti-mouse (CRABP1) or anti-rabbit (CRABP2) secondary systems (DakoCytomation, Carpinteria, CA). Tissues were counterstained with hematoxylin. Cytoplasmic and nuclear staining were scored separately based on the average staining signal intensity throughout the tumor tissue on a scale of 0 (negative), 1 (weak), 2 (moderate) and 3 (strong). Of the 120 tumor samples tested, 105 and 106 had sufficient tissue for analysis of CRABP1 and CRABP2 immunoreactivity, respectively.

### Western blotting

Cytoplasmic and nuclear extracts were prepared according to Dignam *et al.* [36], and whole cell lysates were prepared using Dignam buffer A plus 0.5 % SDS. Cytoplasmic protein (20 μg), nuclear protein (20 μg) and whole cell protein (40 μg) were separated by SDS-polyacrylamide gel electrophoresis and transferred to nitrocellulose membranes by electroblotting. Membranes were immunostained with primary antibodies in 5 % bovine serum albumin (in 1X Tris-buffered saline) at 4 °C overnight. The signal was detected with horseradish peroxidase-conjugated secondary antibodies using the ECL Western Blotting Detection Reagent (GE Healthcare Life Sciences, USA). The following primary antibodies were used for western blot analysis: anti-CRABP1 (1:1,000), anti-CRABP2 (1:1,000), β −actin (Sigma-Aldrich; 1:100,000), α-tubulin (DSHB; 1:10,000) and Lamin A/C (ThermoFisher; 1:1,000).

### Luciferase reporter assay

After siRNA depletion of CRABP1 or CRABP2, MCF-7 cells were seeded in 12-well culture plates at 20,000 cells/well and transfected with the luciferase reporter construct (0.5 μg/well) under the control of a retinoic acid response element (RARE) (pGL3-RARE-luc, Addgene). Alternatively, CRABP1-negative cell lines (BT-549, SK-Br-3, Hs578T) were seeded in 12-well plates, incubated at 37 °C for 24 h and then co-transfected with the CRABP1 expression construct (0.8 μg/well) and pGL3-RARE-Luc (0.5 μg/well) diluted in 250 μL of serum-free medium containing 5 μL PEI. pcDNA3 empty vector served as the negative control for these experiments. Forty-eight h after transfection, cells were treated with RA (in DMSO) at final concentrations of 0, 0.1 and 0.5 μM for 6 h keeping the overall volume of DMSO constant in each well. Cells were then harvested and whole cell lysates prepared using the luciferase cell culture lysis reagent (CCLR, Promega). Luciferase activity was measured with the Luciferase Assay System (Promega) and quantitated using a FLUOstar OPTIMA microplate reader (BMG Labtech) following the manufacturer’s instructions. Triplicate wells were analyzed for each treatment.

### Statistical analysis

Statistical analysis was performed using MedCalc Statistical Software version 12.7.2 (MedCalc Software, Ostend, Belgium) as previously described [25]. Briefly, gene profiling data for CRABP1 and CRABP2 were classified as “low” or “high” by receiver operating characteristic (ROC) curve analysis. Student *t*-test or chi-square test was used to examine the significance of associations between CRABP mRNA levels or immunoreactivity and clinical outcome parameters. Two-way ANOVA was used to test the significance of the effects of siRNA knockdown and RA treatment on cell proliferation. The prognostic significance of CRABP1 and CRABP2 was analyzed by logrank test on Kaplan-Meier survival curves using both gene profiling and TMA immunoassay data.

## Results

### CRABP1 is expressed in ER- and triple-negative breast tumors and is associated with poor clinical outcomes

**Fig. 1:**
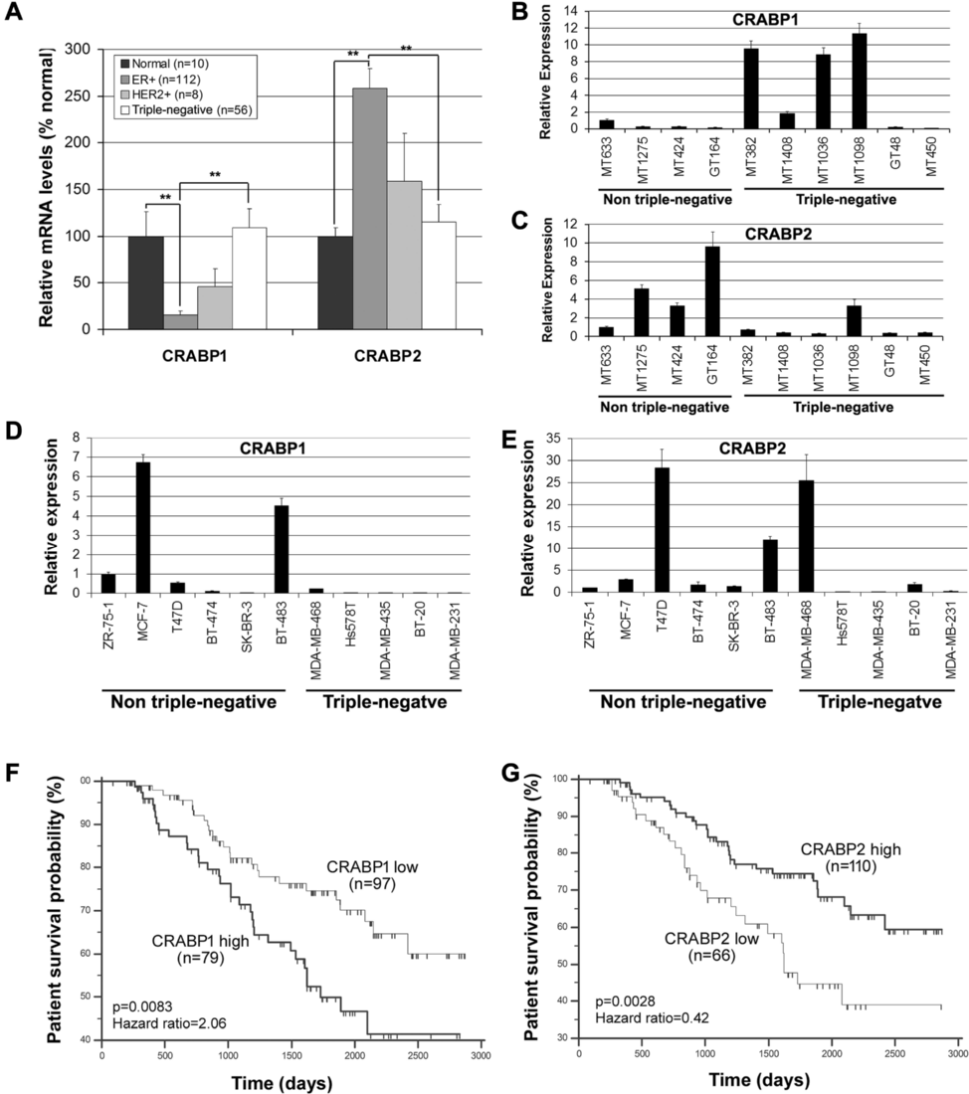
Distinct mRNA expression patterns and prognostic associations of *CRABP1* and *CRABP2* in breast tumors. **a** Comparison of *CRABP1* and *CRABP2* mRNA levels (based on normalized gene microarray signal intensity) in normal mammary tissues (*n* = 10) and human breast cancer subtypes (*n* = 176). **b**, **c** *CRABP1* (**b**) and *CRABP2* (**c**) mRNA levels in tumor tissues sampled from the 176 breast cancer patient cohort. The MT and GT numbers refer to individual patients. RNA levels were measured by real-time quantitative RT-PCR with *GAPDH* serving as the internal control. Expression levels from triplicate reactions are shown relative to MT633. **d**, **e** *CRABP1* (**d**) and *CRABP2* (**e**) mRNA levels in a panel of 11 breast cancer cell lines. RNA levels were analysed by real-time quantitative RT-PCR and shown as fold change relative to ZR-75-1. **f** Kaplan-Meier overall patient survival curves generated based on low and high *CRABP1* mRNA levels determined by ROC analysis. **g** Kaplan-Meier overall patient survival curves generated based on low and high *CRABP2* mRNA levels. n, denotes sample size; *, *p* < 0.05; **, *p* < 0.01

Based on our gene profiling data, *CRABP1* is markedly down-regulated in ER-positive breast tumors in keeping with reports indicating that CRABP1 is silenced in cancer cells [30, 31, 33, 37]. However, *CRABP1* expression is maintained in ER/PR-negative breast tumors compared to normal mammary tissues (Fig. 1a and Table 1). In contrast, *CRABP2* mRNA levels are significantly up-regulated in ER-positive tumors compared to normal and triple-negative tumor tissues (Fig. 1a and Table 1). Neither *CRABP1* nor *CRABP2* levels showed statistically significant difference between HER2-overexpressing tumors and other tissue types. These data indicate that there is an inverse relationship between *CRABP1* and *CRABP2* expression in triple-negative compared to ER/PR-positive tumors

**Table 1:** Clinicopathological associations of CRABP1 and CRABP2 in human breast cancers

	Factor	Classification	cDNA microarray			Tissue microarray									
			n	Geometric mean	p	Cytoplasmic immunoreactivity					Nuclear immunoreactivity				
						Negative		Positive		p	Negative		Positive		p
CRABP1	ER	Negative	64	4.16	**<0.0001**	20		21		**0.0058**	28		13		0.0583
		Positive	112	0.67		49		15			54		10		
	PR	Negative	82	2.80	**<0.0001**	27		25		**0.0040**	37		15		0.1031
		Positive	94	0.67		42		11			45		8		
	Her2	Negative	146	1.39	0.2038	53		31		0.3119	63		21		0.1512
		Positive	30	0.94		16		5			19		2		
	Triple negative	No	120	0.72	**<0.0001**	52		17		**0.0051**	59		10		**0.0142**
		Yes	56	4.63		17		19			23		13		
	Death	No	119	1.13	0.1641	49		18		0.0532	53		9		0.8078
		Yes	57	1.74		20		18			29		14		
	Recurrence	No	88	1.21	0.6020	36		10		**0.0225**	39		7		0.1615
		Yes	88	1.40		33		26			43		16		
	Nodal status	pN0	69	0.81	**0.021**	26		14		1.0000	31		9		1.0000
		pN1-3	107	1.21		42		22			51		14		
	Cancer stage	I	45	1.63	0.1635	21		3		**0.0133**	22		2		0.0919
		II–III	131	1.21		48		33			60		21		
	Nuclear grade	Low	44	0.34	**<0.0001**	20		4		**0.0050**	24		0		**0.0015**
		High	132	2.04		49		32			58		23		
	Mitotic grade	Low	78	0.48	**<0.0001**	36		7		**0.0016**	41		2		**0.0003**
		High	98	2.91		33		29			41		21		
	Arch grade	Low	29	0.76	0.0929	10		0		**0.0145**	10		0		0.1129
		High	147	1.45		59		36			72		23		
	Overall grade	Low	56	0.41	**<0.0001**	25		5		**0.0221**	30		0		**0.0002**
		High	120	2.24		44		31			52		23		
						Cytoplasmic immunoreactivity					Nuclear immunoreactivity				
						0	1	2	3	p	0	1	2	3	p
CRABP2	ER	Negative	64	0.63	**<0.0001**	7	18	13	3	0.0613	5	11	13	12	**0.0010**
		Positive	112	1.37		3	29	19	14		0	6	25	34	
	PR	Negative	82	0.73	**0.0002**	8	23	17	4	**0.0371**	5	12	19	16	**0.0070**
		Positive	94	1.40		2	24	15	13		0	5	19	30	
	Her2	Negative	146	0.95	**0.0369**	9	39	24	13	0.6762	4	15	30	36	0.8405
		Positive	30	1.53		1	8	8	4		1	2	8	10	
	Triple negative	No	120	1.32	**p < 0.0001**	4	30	22	14	0.1545	1	6	28	35	**0.0019**
		Yes	56	0.61		6	17	10	3		4	11	10	11	
	Death	No	119	1.12	0.2089	4	34	21	9	0.1819	1	12	28	27	0.0843
		Yes	57	0.87		6	13	11	8		4	5	10	19	
	Recurrence	No	88	1.02	0.8998	3	26	13	5	0.1837	1	11	18	17	0.1567
		Yes	88	1.04		7	21	19	12		4	6	20	29	
	Nodal status	pN0	69	1.78	0.0819	4	20	14	3	0.2813	2	8	19	12	0.1308
		pN1-3	107	1.07		6	27	18	14		3	9	19	34	
	Cancer stage	I	45	0.81	0.0895	3	13	7	2	0.5660	2	4	11	8	0.5062
		II–III	131	1.12		7	34	25	15		3	13	27	38	
	Nuclear grade	Low	44	1.12	0.5981	1	12	7	4	0.7638	1	2	11	10	0.5616
		High	132	1.05		9	35	25	13		4	15	27	36	
	Mitotic grade	Low	78	1.11	0.4743	2	19	13	10	0.2547	0	4	17	23	0.0618
		High	98	0.98		8	28	19	7		5	13	21	23	
	Arch grade	Low	29	1.20	0.4399	1	6	3	1	0.8747	0	1	3	7	0.5014
		High	147	1.00		9	41	29	16		5	16	35	39	
	Overall grade	Low	56	1.04	0.9369	1	15	10	5	0.5695	0	3	14	14	0.2344
		High	120	1.03		9	32	22	12		5	14	24	32	

Next, we analysed *CRABP* expression in a sampling of ten cDNA samples from patient breast tumor tissues using real-time qRT-PCR analysis. *CRABP1* mRNA was detected at elevated levels (~2 to 11-fold relative to the first tumor sample which was set at 1) in 4 out of 6 triple-negative tumors; however, none of the four non-triple negative tumors showed relative levels larger than 1 (Fig. 1b). In contrast, *CRABP2* mRNA was found at higher levels in non-triple-negative tumors (~3 to 9-fold relative to MT633) compared to triple-negative tumors (with 1 out of 6 tumors showing relative levels higher than 1) (Fig. 1c).

We also screened a panel of 11 breast cancer cell lines for *CRABP1* and *CRABP2* expression. *CRABP1* was detected at elevated levels (4.5–6.8-fold relative to ZR-75-1 which was set at 1) in two non-triple-negative breast cancer cell lines (MCF-7 and BT-483), with none of the five triple-negative cell lines having levels higher than 1 (Fig. 1d). These results suggest that CRABP1 expression is not retained in triple-negative breast cancer cells under *in vitro* growth conditions, perhaps as a consequence of changes in the microenvironment [38]. A similar phenomenon has been reported for other proteins and cancers, including loss of glial fibrillary acidic protein and brain fatty acid-binding protein in malignant glioma cell lines compared to tumor tissue [39–41]. *CRABP2* was expressed at elevated levels (2–28-fold relative to ZR-75-1) in all six non-triple-negative breast cancer cell lines, while only two out of five triple-negative cell lines had relative *CRABP2* levels higher than 1 (Fig. 1e).

We then examined the association of *CRABP* mRNA levels in primary breast tumors with major clinicopathological parameters (Table 1). High *CRABP1* mRNA levels correlated with positive nodal status (*p* = 0.021), high nuclear grade (*p* < 0.0001), mitotic grade (*p* < 0.0001) and overall histological tumor grade (*p* < 0.0001) (Table 1). Such correlations were not observed with *CRABP2* mRNA levels. Kaplan-Meier survival analysis further showed that high levels of *CRABP1* mRNA were significantly (*p* = 0.0083) associated with lower patient survival probability (Fig. 1f), whereas high levels of *CRABP2* mRNA were significantly associated with better prognosis (*p* = 0.0028; Fig. 1g). Again, these results suggest opposing roles for CRABP1 and CRABP2 in breast cancer clinical outcomes.

### Subcellular localization of CRABP1 determines prognostic significance

**Fig. 2:**
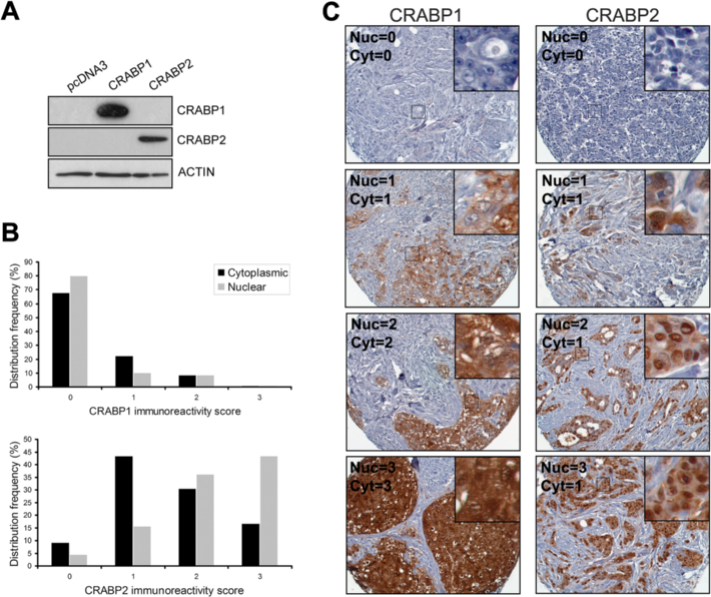
Immunoreactivity and subcellular distribution of CRABP1 and CRABP2 in a human primary breast tumor TMA. **a** Western blot of CRABP1 and CRABP2 in MDA-MB-435 cells transfected with a CRABP1 or CRABP2 expression construct, respectively. **b** Frequency of breast tumors with different subcellular immunoreactivity scores for CRABP1 and CRABP2: 0, negative; 1, weak; 2, intermediate; 3, strong. **c** Selected tissue sections from a human breast cancer TMA immunostained with anti-CRABP1 and anti-CRABP2 antibodies. Nuclear (Nuc) and cytoplasmic (Cyt) scores are indicated

CRABPs serve as intracellular chaperones for RA and modulate its nuclear availability and biological activity [42, 43]. The subcellular localization of CRABPs is therefore a critical determinant of their function. To investigate whether there is any association between the subcellular distribution of CRABP1 and 2 and clinical outcomes, we conducted immunohistochemical analysis of a TMA containing primary tumor samples from 120 breast cancer patients. We first validated the specificity of our anti-CRABP1 and anti-CRABP2 antibodies by western blot analysis of whole cell lysates prepared from MDA-MB-435 cells (negative for both CRABP1 and 2) transfected with CRABP1 or CRABP2. No cross-immunoreactivity was observed for these two antibodies (Fig. 2a).

We found that 34.3 % and 21.9 % of the scored tumors showed positive cytoplasmic and nuclear CRABP1 immunoreactivity, respectively (Fig. 2b and Table 1). TMA tissues with different CRABP1 and CRABP2 cytoplasmic and nuclear immunostaining intensities are shown in Fig. 2c. Elevated levels of CRABP1 in the cytoplasm, but not in the nucleus, was associated with negative ER (*p* = 0.0058) and PR status (*p* = 0.004). Both cytoplasmic and nuclear CRABP1 immunoreactivities were positively correlated with triple-negative status and high histological tumor grade (Table 1).

In contrast to CRABP1, the great majority of breast tumor tissues scored positive for CRABP2, with 90.7 % and 95.4 % of the scored samples showing cytoplasmic and nuclear CRABP2 immunoreactivity, respectively (Fig. 2b and Table 1). Close to half (43.5 %) of the tumors showed strong nuclear CRABP2 immunoreactivity (score = 3), with a strong cytoplasmic CRABP2 signal observed in 16.7 % of tumors (Fig. 2b–c). Nuclear CRABP2 protein levels were positively correlated with positive ER (*p* = 0.001), PR (*p* = 0.007) and overall non-triple negative status (*p* = 0.0019). A correlation was also observed between high cytoplasmic CRABP2 levels and positive PR status (*p* = 0.0371, Table 1). No correlations with other clinicopathological parameters were observed for both cytoplasmic and nuclear CRABP2 immunoreactivity (Table 1).

**Fig. 3:**
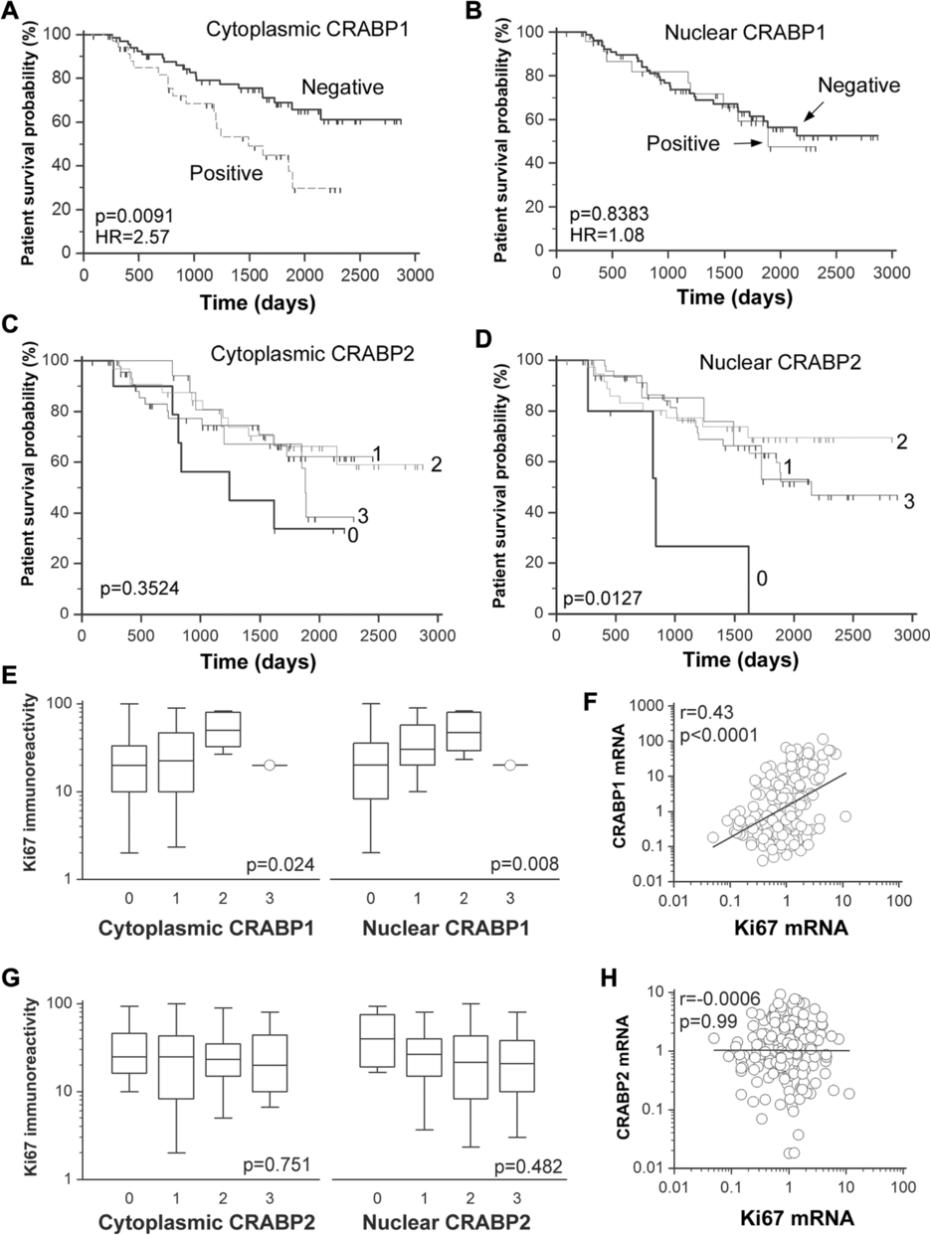
Associations of subcellular CRABP1 and CRABP2 levels with breast cancer patient survival and Ki67 immunoreactivity. CRABP1, CRABP2 and Ki67 protein levels were determined by immunohistochemical analysis of a TMA containing triplicate cores for each tumor from a 120 primary breast tumor cohort. **a**-**b** Association of cytoplasmic and nuclear CRABP1 levels with patient survival. **c**-**d** Association of cytoplasmic and nuclear CRABP2 levels with patient survival. **e** Positive correlation of cytoplasmic and nuclear CRABP1 protein levels with Ki67 immunoreactivity. **f** Positive correlation of *CRABP1* and *Ki67* mRNA levels based on gene microarray analysis. **g** No significant correlation was observed between subcellular CRABP2 levels and Ki67 immunoreactivity. **h** No correlation was evident between *CRABP2* and *Ki67* mRNA levels. HR, hazard ratio; p, statistical significance level; r, correlation coefficient. Scores: 0, negative; 1, weak; 2, intermediate; 3, strong. As the size of samples expressed CRABP1 is small in our TMAs, tumor samples were classified into “positive” and “negative” groups for survival analysis

We then correlated subcellular levels of CRABPs with patient survival probabilities. We found that patients with tumors that were positive for cytoplasmic, but not nuclear, CRABP1 had a significantly lower patient survival probability (*p* = 0.0091) (Fig. 3a–b). In contrast, nuclear, but not cytoplasmic, CRABP2 immunoreactivity was positively associated with patient survival probability (*p* = 0.0127) (Fig. 3c–d). Thus, our TMA analysis shows that: (i) the clinicopathological associations of CRABP1 and 2 at the protein level are in good agreement with that of the gene profiling data, (ii) CRABP1 is more frequently found in the cytoplasm whereas CRABP2 is more abundant in the nucleus of primary breast tumors, and (iii) clinical outcome and prognostic implications can be attributed to cytoplasmic CRABP1 and nuclear CRABP2, suggesting distinct subcellular functions.

### CRABP1 correlates with tumor cell proliferation and is a potent inhibitor of RA signalling

To further understand the opposing roles of CRABP1 and CRABP2 in breast cancer progression, we immunostained our breast cancer TMA with Ki67, a cell proliferation marker (Additional file 1: Figure S1), and examined its correlation with CRABP protein levels. We found that both cytoplasmic (*p* = 0.024) and nuclear (*p* = 0.008) CRABP1 levels were positively correlated with Ki67 immunoreactivity (Fig. 3e). In contrast, neither cytoplasmic nor nuclear CRABP2 levels correlated with Ki67 protein levels (Fig. 3g). As expected, the mRNA levels of *Ki67* also showed significant positive correlation with that of *CRABP1*, but not *CRABP2* mRNA, based on gene profiling analysis (Fig. 3f, h). These results suggest that CRABP1 may affect breast cancer progression by enhancing tumor cell proliferation through modulation of RA signalling. The lack of correlation between CRABP2 and Ki67 suggests that the effect of CRABP2 on patient survival may be mediated through differentiation and apoptosis rather than proliferation, as previously reported for APL [17, 44].

**Fig. 4:**
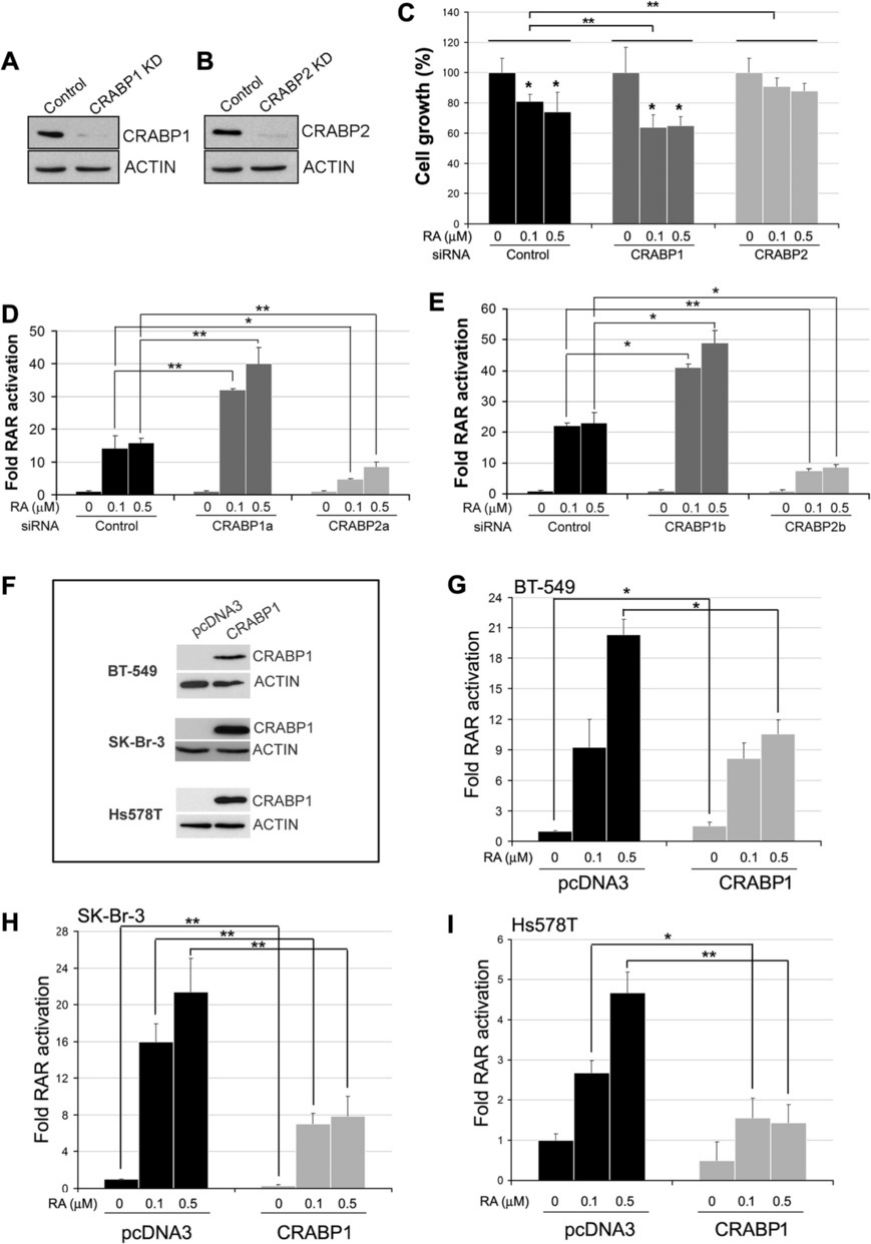
CRABP1 and CRABP2 differentially modulate RA-induced growth inhibition and RAR transcriptional activity. **a**, **b** Western blot analysis of MCF-7 cells transfected with *CRABP1* or *CRABP2* siRNAs using antibodies against CRABP1 or CRABP2. **c** Relative growth rate of MCF-7 cells treated with the indicated concentrations of RA after transfection with non-specific (control) and specific siRNAs targeting CRABP1 or CRABP2. Significance of difference was tested using two-way ANOVA. **d** RAR transactivation (measured by luciferase activity) of CRABP1a-depleted or CRABP2a-depleted MCF-7 cells transfected with a luciferase reporter construct under the control of a RARE. Cells were treated with DMSO (vehicle control) or RA for 6 h before harvest. Luciferase activity (a measure of RAR activation) is shown as fold change relative to cells that were cultured in the absence of RA. **e** The luciferase assay was repeated with a second set of siRNAs targeting CRABP1 and CRABP2 (CRABP1b, CRABP2b). **f** Western blots showing ectopic expression of CRABP1 in three human breast cancer cell lines. **g**, **h**, **i** The effects of ectopic expression of CRABP1 on RAR transactivation (measured by luciferase activity) were examined in BT-549 (**g**), SK-Br-3 (**h**) and Hs578T (**i**) cells co-transfected with a CRABP1 cDNA construct and a RARE-luciferase reporter construct. Cells were treated with RA for 6 h at the indicated concentrations. Luciferase activity (a measure of RAR activation) was adjusted based on protein concentrations of individual lysates and shown as fold change relative to control cells transfected with empty vector and cultured in the absence of RA. KD, denotes knockdown; *, *p* < 0.05; **, *p* < 0.01

CRABP1 and CRABP2 may play distinct roles in RA/RAR-mediated transcriptional activity [42, 45]. To investigate the role of CRABP1 in RA action, we used siRNAs to deplete the RA-responsive MCF-7 cells of either CRABP1 or CRABP2 (Fig. 4a–b). A small but statistically significant increase in growth inhibition was observed when CRABP1-depleted cells were treated with RA, with control cells showing 81 and 74 % relative growth at 0.1 μM and 0.5 μM RA, respectively, and CRABP1-depleted cells showing 65 and 66 % relative growth at 0.1 μM and 0.5 μM RA, respectively (Fig. 4c). A stronger growth inhibition effect was observed upon treatment of CRABP1-depleted cells with RA in the absence of serum (Additional file 1: Figure S2). In contrast to CRABP1, knockdown of CRABP2 had little effect on RA-mediated cell growth inhibition (91 % at 0.1 μM and 88 % at 0.5 μM) (Fig. 4c).

The growth inhibitory effect of RA is believed to be mediated through activation of RARs [23, 24, 45]. We therefore examined the effect of CRABP1 and CRABP2 on RA-induced RAR activation using the luciferase reporter system. We used two different siRNAs to deplete each of the two *CRABP* genes in MCF-7 cells. CRABP-depleted MCF-7 cells were then transfected with a RA-responsive RARE-luciferase vector and treated with RA. An RA dose-dependent induction in luciferase activity (indicative of RAR activation) was observed for both control and siRNA transfected cells (Fig. 4d–e). However, CRABP1 depletion resulted in a ~2-fold increase in RAR activation in the presence of RA compared to control cells, whereas depletion of CRABP2 resulted in decreased RAR/RA-mediated transcriptional activity (Fig. 4d–e). These results suggest that in contrast to CRABP2 which plays a positive role in RAR activation, CRABP1 may serve as an inhibitor of RA signalling in breast cancer cells.

We then co-transfected two triple-negative breast cancer cell lines (Hs-578 T and BT-549) and one ER-negative/HER2 overexpressing cell line (SK-Br-3) with a CRABP1 expression construct and the RARE-luciferase reporter vector. CRABP1 expression in transfected cells was verified by western blotting (Fig. 4f). Endogenous CRABP1 was not detected in any of these three cell lines. Luciferase activity was induced by RA in all three cell lines in a dose-dependent manner in cells transfected with empty vector (Fig. 4g–i). A significant reduction in RA-induced RAR activation was observed in all three cell lines upon transfection with the CRABP1 expression construct. Specifically, SK-Br-3 and Hs578T cells showed reduced RAR-activation in both 0.1 μM and 0.5 μM RA-treated cells upon CRABP1 expression (Fig. 4h–i). Reduced luciferase activity was only observed at the highest concentration of RA tested in BT-549 cells (Fig. 4g).

**Fig. 5:**
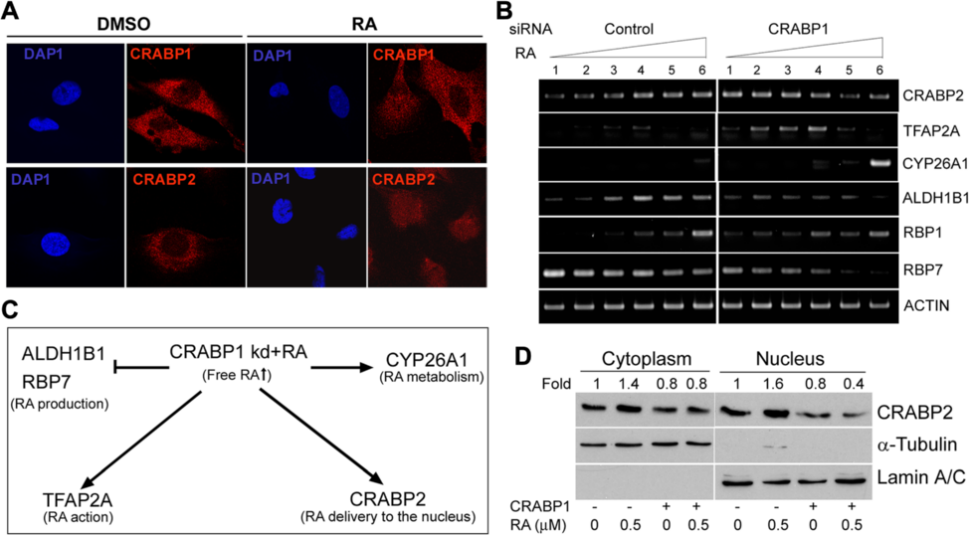
**a** Subcellular localization of CRABP1 and CRABP2 in MCF-7 cells treated with RA. Cells were cultured in medium with serum for 24 h and then treated with 0.5 μM RA in serum-free medium for 6 h. An equivalent amount of DMSO was added to control cells. Cells were immunostained with anti-CRABP1 (red, upper panel) or anti-CRABP2 (red, lower panel) antibodies as described in Materials and Methods. DAP1 (blue) staining was used to visualize the nucleus. **b** Expression of RA-responsive genes in MCF-7. MCF-7 cells underwent two rounds of transfection with scrambled or *CRABP1* siRNAs. Cells were treated with increasing concentrations of RA [lanes 1 to 6 (0, 5 × 10^−5^, 5 × 10^−4^, 5 × 10^−3^, 5 × 10^−2^, 5 × 10^−1^ μM RA)]. RNA was purified from each culture and semi-quantitative RT-PCR was carried out using gene-specific primers (Additional file 1: Table S1). **c** Summary of the effect of CRABP1 and RA on downstream genes and pathways. **d** Western blots showing the subcellular distribution of CRABP2 in SK-Br-3 cells upon CRABP1 overexpression and RA treatment. Densitometric analysis was used to quantitate CRABP2 signal intensity in the cytoplasm and nucleus relative to the cytoplasmic marker (β-tubulin) and nuclear marker (lamin A/C), respectively. Changes in band intensities are shown as fold change in relation to lane 1 (for cytoplasmic CRABP2) and lane 5 (for nuclear CRABP2)

Next, we analysed CRABP1 and CRABP2 subcellular localization in response to RA in MCF-7 cells. Both proteins were primarily found in the cytoplasm in the absence of RA (Fig. 5a). In contrast to CRABP2 which translocated to the nucleus upon RA treatment, there was no change in CRABP1 subcellular localization upon RA treatment (Fig. 5a). These results are consistent with a general role for CRABP1 in sequestering RA in the cytoplasm, rendering it unavailable for RAR activation.

### CRABP1 attenuates RA signalling through modulation of the expression of RA responsive genes

To further explore the mechanism involved in CRABP1/RA-mediated effects in breast cancer cells, we examined the expression of critical genes implicated in RA synthesis, trafficking, metabolism and action upon CRABP1 manipulation and RA treatment. MCF-7 cells were transfected with scrambled or *CRABP1* siRNAs and treated with increasing amounts of RA. Levels of *CRABP2*, a facilitator of RA signalling to the nucleus [46], were upregulated upon CRABP1 depletion in the absence of RA (Fig. 5b). Upon RA treatment, *CRABP2* levels were induced in control cells in a dose-dependent manner but not in *CRABP1*-depleted cells. The transcription factor AP-2α (*TFAP2A*), a RA-inducible gene with tumor-suppressing activity [47, 48], was upregulated by CRABP1 depletion and RA treatment at a dose up to 5 nM. The expression of the RA metabolizing enzyme *CYP26A1* was induced at high concentrations of RA (500 nM RA) in both control and *CRABP1*-depleted cells, with enhanced up-regulation observed in the latter. The observation that *CYP26A1* levels are up-regulated to a higher degree in *CRABP1*-depleted compared to control cells argues against the idea that CRABP1 promotes RA metabolism [49–51]. The expression of aldehyde hydrogenase *ALDH1B1* gene, which is involved in RA biosynthesis and associated with cancer cell stemness [52], was induced by RA in the control cells but not in the *CRABP1*-depleted cells. Interestingly, the two cellular retinol-binding protein genes (*RBP1* and *RBP7*), encoding proteins that facilitate retinol uptake, storage and/or metabolism [53–56], exhibited opposite expression patterns upon *CRABP1* knockdown and RA treatment, with *RBP1* being induced by RA in both control cells and *CRABP1*-depleted cells, and *RBP7* being down-regulated by CRABP1 depletion and suppressed by RA in a dose-dependent manner. These results (summarized in Fig. 5c) indicate that CRABP1 has an inhibitory effect on RA action through modulation of a spectrum of genes involved in various aspects of the RA network including RA biosynthesis, metabolism and intracellular trafficking.

Next, we examined the effect of CRABP1 expression on the nuclear translocation of CRABP2, which is an important indication of RA signalling to the nucleus [57]. SK-Br-3 cells were transfected with a CRABP1 expression construct and treated with RA. We observed higher levels of cytoplasmic and nuclear CRABP2 upon RA treatment in control cells (Fig. 5d). Furthermore, a marked decrease in nuclear CRABP2 levels was observed in CRABP1-overexpressing cells upon RA treatment. Taken together, these results suggest that CRABP1 acts as an inhibitor of RA action by restricting RA access to the nucleus through down-regulation of nuclear CRABP2 levels.

**Fig. 6:**
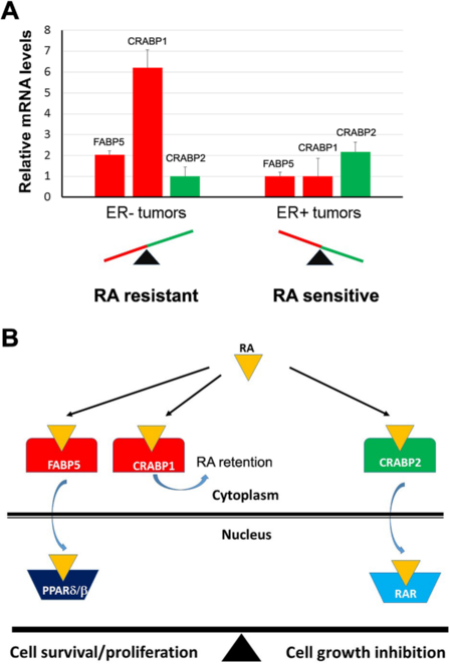
Schematic representation of the effects of different RA binding proteins on modulation of RA action in breast cancer. **a** Relative mRNA levels of *FABP5*, *CRABP1* and *CRABP2* in ER-negative (*n* = 64) and ER-positive (*n* = 112) primary breast cancer tissue samples. The mRNA levels for each gene were determined based on the normalized signal intensity of the gene microarray data and are shown relative to *CRABP2* (set as 1) in the case of ER-negative tumors and *FABP5* (set as 1) in the case of ER-positive tumors. These data provide insight as to the possible underlying cause of RA resistance in ER-negative tumors. **b** A schematic model illustrating the distinct roles of CRABP1, CRABP2 and FABP5 in modulating cellular response to RA in breast cancer cells. FABP5 channels RA to PPARδ/β, a nuclear receptor which promotes cell proliferation. CRABP1 sequesters RA in the cytoplasm. CRABP2 delivers RA to RAR, leading to cell growth inhibition. The balance between FABP5/CRABP1 and CRABP2 expression levels determines the cellular response to RA (cell growth promotion or inhibition)

Our results suggest that CRABP1, in addition to the previously identified FABP5 and CRABP2, is a key factor regulating breast cancer cell response to RA. To explore the possibility that expression levels of these three RA binding proteins might be useful predictors of primary breast tumor sensitivity to RA, we analyzed their relative mRNA levels in our 176 breast cancer patient cohort based on gene microarray analysis. *CRABP1* and *FABP5*, encoding RA binding proteins associated with RA resistance, were 5.2- and 1-fold higher, respectively, in ER-negative breast tumors compared to ER-positive tumors. On the other hand, levels of *CRABP2*, a positive modulator of RA signalling and activity, were 1.2-fold higher in ER-positive compared to ER-negative tumors (Fig. 6a). These results suggest that CRABP1 in particular may play an important role in the RA resistance observed in ER-negative tumors.

## Discussion

Cellular response to RA is believed to depend on two different classes of nuclear receptors, RARs and PPARs [23, 24]. RAR activation by RA results in cell growth inhibition whereas PPARδ/β activation stimulates cell proliferation. These two RA signaling pathways are in turn modulated by two intracellular RA binding proteins: CRABP2 which channels RA to the nucleus to target RAR, and FABP5 which delivers RA to the nucleus thereby activating PPARδ/β [23, 24]. We previously showed that FABP5 is preferentially expressed in ER- and triple-negative breast cancers, molecular subtypes believed to be resistant to RA treatment [25]. High levels of FABP5, as well as a low ratio of CRABP2 to FABP5, are associated with poor prognosis. In this study, we identify a third RA-binding protein, CRABP1, as an inhibitor of RA action and an adverse factor for clinical outcome in breast cancer. Like FABP5, CRABP1 is preferentially expressed in ER- and triple-negative breast cancer. We propose a model whereby CRABP1 can compensate or synergize with FABP5 to compete with CRABP2 for RA, by sequestering RA in the cytoplasm, thereby reducing RA access to RAR (Fig. 6b).

The role of CRABP1 in carcinogenesis and tumor progression is poorly understood and contradictory. For example, CRABP1 is down-regulated in some human cancers and cell lines [31–33], with DNA methylation proposed to contribute to CRABP1 silencing [33, 58–60]. DNA methylation-mediated silencing of CRABP1 has been observed in a subset of breast carcinoma tissues [60]. CRABP1 has been proposed to be a tumor suppressor in esophageal squamous cell carcinoma, with reduced CRABP1 levels associated with increased cell growth and distant lymph node metastasis [33]. Reduced levels of CRABP1 are also associated with poorer prognosis in serous (*n* = 40) and clear cell ovarian adenocarcinoma (*n* = 59) [31]. On the other hand, high levels of CRABP1 have been linked to lymph node metastasis and poor differentiation/high grade in pancreatic neuroendocrine tumors [30].

In agreement with a pro-carcinogenic role for CRABP1, we found an association between CRABP1 expression and worse clinical outcomes in breast cancer using both gene profiling and TMA analysis. Similar to a previous report indicating that CRABP1 is differentially expressed in different subtypes of pituitary adenomas [37], we found that CRABP1 is downregulated in ER+ breast tumors, but expressed in ER- and triple-negative tumors. These results suggest that downregulation of CRABP1 expression in cancer cells may be modulated by specific signaling pathways in different cancer subtypes (e.g. estrogen signalling), and that its role in tumor progression may differ between cancer types. It is noteworthy that estrogen signaling has been linked to the regulation of DNA methylation, perhaps explaining to some extent the reduced expression of CRABP1 observed in ER+ compared to ER- breast cancers [61].

CRABP1 has the highest RA binding affinity of all RA binding proteins [42]. It is generally believed that CRABP1 represses cellular response to RA by sequestering RA and/or promoting RA catabolism, reducing its availability in the nucleus for activation of RARs [51, 62–64]. Several studies have demonstrated that CRABP1 promotes RA metabolism [49–51]. However, the fact that RA metabolites can activate RAR *in vitro* [65], combined with the observation that a positive relationship exists between RA metabolism and cell growth inhibition in several cancer cell lines [66], suggest that CRABP1-mediated RA metabolism may not account for RA resistance. In light of our observations that: (i) cytoplasmic CRABP1 in breast tumors is an adverse factor in clinical outcomes and (ii) CRABP1 accumulates in the cytoplasm of cells treated with RA, we propose that the primary role of CRABP1 in breast cancer is to sequester RA in the cytoplasm, thereby preventing RAR activation in the nucleus (Fig. 6b). The up-regulation of the RA-metabolizing gene *CYP26A1* observed upon CRABP1 depletion at high RA concentration further suggests a negative association between CRABP1 and RA metabolism.

Over five hundred genes are known to be regulated by RA, including *CRABP2* which has a functional RARE in its promoter region [67, 68]. CRABP2 serves as a positive regulator of RA signalling in breast cancer cells [23, 24, 42] and its expression can be induced by RA in various cell types [67–69]. In this study, we found that CRABP1 and CRABP2 have inverse expression patterns in breast tumors and play an opposing role in the mediation of RA action in breast cancer cells. We further report that CRABP1 negatively regulates *CRABP2* expression. While increased CRABP2 expression has been observed in AB1 embryonic stem cells with homozygous deletion of *CRABP1* [70], this is the first report demonstrating that CRABP1 has an inhibitory effect on CRABP2 expression in cancer cells. Intriguingly, CRABP1 not only inversely regulates *CRABP2* expression, but also affects nuclear translocation of CRABP2 in breast cancer cells. We postulate that CRABP1 plays a key role in attenuating RA activity in breast cancer cells, with high levels of CRABP1 reducing availability of RA in the nucleus. In turn, RA sequestration to the cytoplasm represses RA-mediated nuclear translocation of CRABP2 and induction of CRABP2 expression.

In addition to *CRABP2*, our data indicate that CRABP1 modulates the expression of various genes implicated in RA biosynthesis, metabolism and action. With the exception of *RBP7*, all these genes have previously been shown to be RA-regulated [68]. We observed that the genes encoding CYP26A1 (catalyzes RA metabolism) and ALDH1B1 (catalyzes RA biosynthesis) are up-regulated and down-regulated, respectively, in the presence of RA upon CRABP1 depletion. We speculate that this is due to a feedback process as CRABP1 depletion may increase levels of free RA (especially when cells are exposed to high doses of RA), in turn resulting in accelerated RA metabolism and reduced RA synthesis. Therefore, CRABP1 may play a role in regulating cellular levels of free RA. RBP1 and RBP7 are retinol binding proteins which facilitate retinol storage and/or retinol-RA conversion [53–56]. The different expression patterns observed for these two genes in control and CRABP1-depleted MCF-7 cells suggests opposite roles. We propose that *RBP1* (upregulated by RA but not affected by CRABP1) may be involved in retinol storage whereas *RBP7* (down-regulated by RA and CRABP1 depletion) may be involved in retinol metabolism producing RA.

*TFAP2A* is a RA-inducible gene whose expression increased upon CRABP1 knockdown in MCF-7 cells. *TFAP2A* encodes AP-2α, recently shown to be essential for RA action [71], has previously been reported to stimulate CRABP2 expression in mammary epithelial cells and breast cancer cells [72]. AP-2α significantly enhances RA-induced RAR activation in breast cancer cells (our unpublished data). These combined data suggest the presence of a CRABP1-AP-2α-CRABP2 axis which modulates RA action in breast cancer cells.

In summary, we show that CRABP1 expression is maintained in ER- and triple-negative breast tumors, and that elevated levels of CRABP1 is a significant indicator of high tumor grade, Ki67 immunoreactivity, and poor prognosis. Our data indicate that cytoplasmic CRABP1, like FABP5, is a potent inhibitor of RA signalling. Elevated levels of CRABP1 may lead to RA resistance in breast cancer cells through sequestration of RA in the cytoplasm thereby preventing RA-mediated induction of RAR. We further demonstrate that CRABP1 attenuates RA activity by modulating the expression of important RA-regulated genes implicated in cellular RA availability, traffic and action. Thus, both CRABP1 and FABP5 represent potential therapeutic targets to overcome RA resistance in breast cancer. The discovery that there are at least three proteins involved in RA transport in breast cancer cells (CRABP1, CRABP2 and FABP5 [23–25]), helps to address the molecular mechanism governing RA resistance in ER-negative or triple-negative breast cancer, and provides molecular tools to predict and eventually overcome RA resistance in breast cancer prevention and therapy.

## Additional file

Additional file 1: Table S1. Nucleotide sequences of PCR primers. **Figure S1.** Sample images of Ki67 immunohistochemical staining of a human breast cancer TMA. The percentages shown are the averages of the percent Ki67 positive cells in three TMA cores of the same tumor. Tumor ID numbers are indicated at the bottom. **Figure S2.** Effect of CRABP1 expression on RA-induced cell growth inhibition in MCF-7 cells. Cells were transfected with siRNA targeting CRABP1 (CRABP1 kd) or scrambled siRNA (control). Transfected cells were then seeded in a 96-well plate, cultured for 24 h and then treated with RA in serum-free medium at the indicated concentrations for two days. Cell proliferation was analyzed using the MTS cell proliferation assay system (Promega) following the manufacturer’s protocol. Significance of difference was analyzed by two-way ANOVA. *denotes *p* < 0.05 and ***p* < 0.01.

## Abbreviations

ALDH1B1: Aldehyde dehydrogenase 1 family, member B1
APL: Acute promyelocytic leukemia
CRABP: Cellular retinoic acid-binding protein
CYP26A1: Cytochrome P450, family 26, subfamily A, polypeptide 1
DMEM: Dulbecco’s Modified Eagle’s medium
ER: Estrogen receptor
HER2: Epidermal growth factor receptor 2
PEI: Polyethylenimine
PPAR: Peroxisome proliferator-activated receptor
PR: Progesterone receptor
RA: Retinoic acid
RAR: Retinoic acid receptor
RARE: Retinoic acid response element
RBP: Retinol binding protein
RXR: Retinoid-X-receptor
TFAP2A: Transcription factor AP-2 alpha
