# Supplementary material for: CRABP1 is associated with a poor prognosis in breast cancer: adding to the complexity of breast cancer cell response to retinoic acid
Source: Mol Cancer. 2015 Jul 5;14:129. doi: 10.1186/s12943-015-0380-7 (PMC4491424; doi:10.1186/s12943-015-0380-7)

Additional file 1: Table S1. Nucleotide sequences of PCR primers.

Additional file 1: Figure S1. Sample images of Ki67 immunohistochemical staining of a human breast cancer TMA. The percentages shown are the averages of the percent Ki67 positive cells in three TMA cores of the same tumor. Tumor ID numbers are indicated at the bottom.

Additional file 1: Figure S2. Effect of CRABP1 expression on RA-induced cell growth inhibition in MCF-7 cells. Cells were transfected with siRNA targeting CRABP1 (CRABP1 kd) or scrambled siRNA (control). Transfected cells were then seeded in a 96-well plate, cultured for 24 h and then treated with RA in serum-free medium at the indicated concentrations for two days. Cell proliferation was analyzed using the MTS cell proliferation assay system (Promega) following the manufacturer’s protocol. Significance of difference was analyzed by two-way ANOVA. * denotes $p<0.05$ and ** $p<0.01$.

Additional file 1: Table S1. Nucleotide sequences of PCR primers

CRABP1	Forward	5'-TTGCGAGCTCAGAGTGTGC-3′
	Reverse	5'-ATTGTCATGGGGAAAACGGG-3′
CRABP2	Forward	5'-TGCTGAGGAAGATTGCTGTG-3′
	Reverse	5'-TCTTTGTTGGTGTAGGGGAG-3′
TFAP2A	Forward	5'-CTGGGCACTGTAGGTCAAT-3′
	Reverse	5'-GAAGACTTCGTTGGGGTTC-3′
CYP26A1	Forward	5'-TGGCTGCCTCTCTAACCTG-3′
	Reverse	5'-GATCTTGGCGCGAATGTTC-3′
ALDH1B1	Forward	5′-GAACCCAAGCGTGATCCTG-3′
	Reverse	5′-CCTAGTCCAAGGCGTAAGAC-3′
RBP1	Forward	5'-TCCAGTCACTCCCCGAAATG-3
	Reverse	5'-TCCAGTCACTCCCCGAAATG-3'
RBP7	Forward	5'-CTTCGAGGGCTACATGCTG-3′
	Reverse	5'-GAGTCCTCGTCGCCAAATG-3′
CCND1	Forward	5′-TGCCAACCTCCTCAACGAC-3′
	Reverse	5′-CCAGGTTCCACTTGAGCTTG-3′
ACTIN	Forward	5'-CTGGCACCACACCTTCTAC-3'
	Reverse	5'-CATACTCCTGCTTGCTGATC-3'

Additional file 1: Figure S1

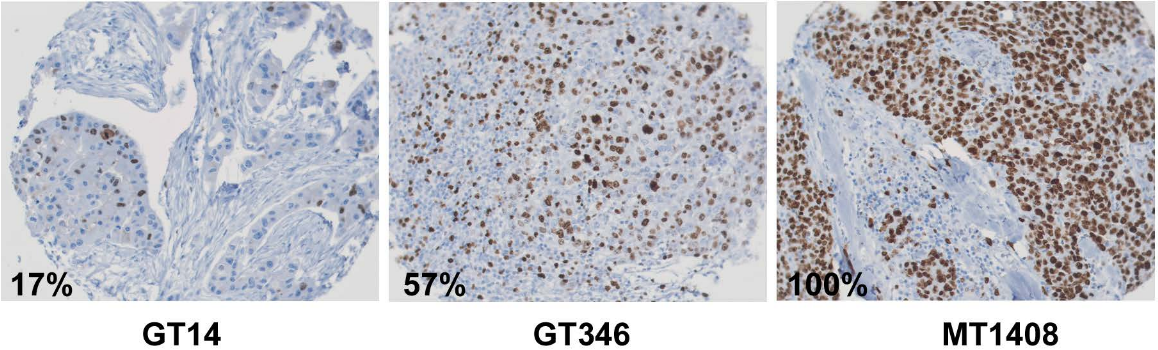

Additional file 1: Figure S2

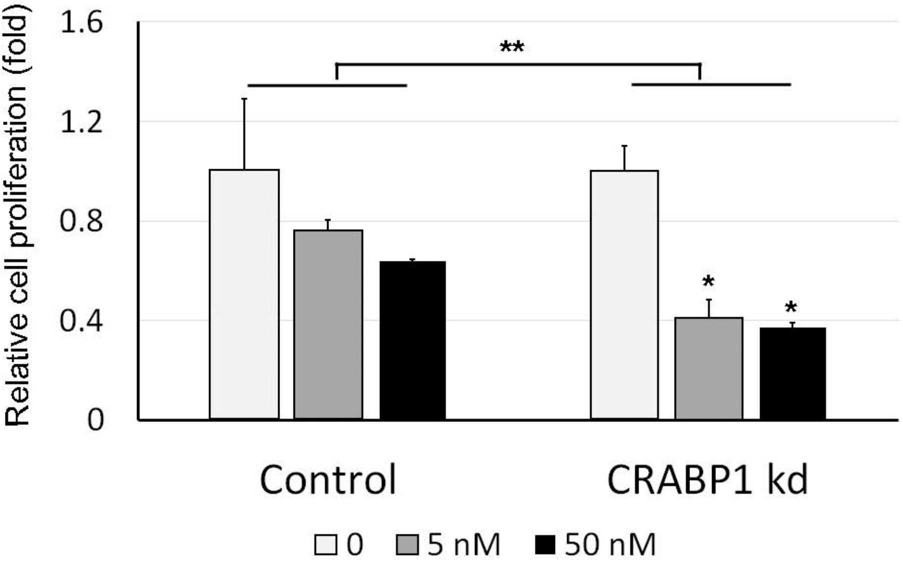

Supplement: Additional file 1: Table S1. — Nucleotide sequences of PCR primers. Figure S1. Sample images of Ki67 immunohistochemical staining of a human breast cancer TMA. The percentages shown are the averages of the percent Ki67 positive cells in three TMA cores of the same tumor. Tumor ID numbers are indicated at the bottom. Figure S2. Effect of CRABP1 expression on RA-induced cell growth inhibition in MCF-7 cells. Cells were transfected with siRNA targeting CRABP1 (CRABP1 kd) or scrambled siRNA (control). Transfected cells were then seeded in a 96-well plate, cultured for 24 h and then treated with RA in serum-free medium at the indicated concentrations for two days. Cell proliferation was analyzed using the MTS cell proliferation assay system (Promega) following the manufacturer’s protocol. Significance of difference was analyzed by two-way ANOVA. *denotes p < 0.05 and **p < 0.01. [file 12943_2015_380_MOESM1_ESM.pdf]
